# The Preparation and Coloring of Lantern-Slides Suitable for Scientific Demonstrations

**Published:** 1898-01

**Authors:** J. Alfred Scott


### Abstracts and Translations.

---

### THE PREPARATION AND COLORING OF LANTERN-SLIDES SUITABLE FOR SCIENTIFIC DEMONSTRATIONS.

BY J. ALFRED SCOTT, M.A., M.D., F.R.C.S.I.[1]

As the optical lantern is now used so largely in the illustration of communications made before most societies, I may be pardoned for bringing this subject before you, although it is not strictly a subject connected with dentistry. But as there are some simple methods of preparing suitable slides for this purpose, all of which I use for my own lectures, and which are not generally known, I

---

[1] Professor of Physiology, Royal College of Surgeons, Ireland.

thought the subject might have sufficient interest for some to warrant my occupying so much of your time.

At the outset I wish to state that I do not intend to speak of the preparation of slides by purely photographic methods, as these are fully described in the photographic manuals and elsewhere, and indeed, from the beautiful specimens in the museum attached to the meeting, I see that many of you have very little to learn.

There are several ways by which fairly good diagrams can be prepared on plain glass. The glass slips, which must be three and a quarter inches square, may be purchased coated with a soft kind of blue paint on which lines may be etched with a needle, but a method I find to be simpler and more easy to work is to cover the plain glass with a thin deposit of carbon from the flame of a small lump of camphor, or a paraffin lamp from which the chimney has been removed and the wick turned low. A thin layer of carbon which is transparent and brown rather than black will be sufficiently opaque, as the deposit appears more opaque in the lantern than it does when held between the eye and a window. On the smoked surface anything can be written or etched with a point; a needle projecting about half an inch from a pen-handle being very satisfactory. The effect on the screen is similar to chalk lines on a black-board. If the slide is to be carried any distance it should be fixed with some varnish; I generally use gum benzoin dissolved in alcohol (about one ounce to the pint) for this purpose. It will soon dry, and can be protected by another glass of the same size fastened by paper strips round the edges.

Two small pieces of paper about one-eighth of an inch in diameter should be fastened on the front, one piece at each of the upper corners, as a guide in putting the slide into the lantern, as it is sometimes very difficult to say which way a slide belonging to another person should be placed when seen for the first time in the very feeble light when the gas is turned down. To repeat, when the writing or drawing is held in the hand, and appears right side up, the two spots should be visible on the upper corners.

Muffed or obscured glass can be purchased cut three and a quarter inches square. Some (the common variety) has a rather coarse grain, some has a very fine grain; this latter should be procured. It is stocked by most photographic dealers. On this surface a hard lead-pencil works very nicely. The glass is sufficiently transparent to enable a drawing previously made on paper or in a book to be traced, but is too opaque for the lantern unless it be varnished. For this purpose a thin solution of Canada balsam

(preferably the dried form used in histology) dissolved in turpentine or xylol can be poured over the muffed surface. It should be dried with a little extra heat, say by holding it before the fire until the glass is warm and testing if it be sticky when cold. This varnish, being nearly of the same refractive index as glass, obliterates the surface, leaving the pencil lines on apparently transparent glass. The slide should then be mounted and "spotted" as described above.

Various colored inks may be easily made, which will write on unprepared glass, by dissolving about ten per cent. of dextrin in a solution of any of the aniline dyes. Eosin and methyl or malachite green are strongly contrasting colors and suitable for this purpose,—they should, however, not be mixed. Indian-ink similarly thickened is very useful for outlines or writing lists of tabulated matter, etc., the lines or principal words being put in with the colored inks. These solutions in dextrin can also be used with a brush either pure or diluted to cover large surfaces on the plain glass.

If the glass be previously coated with a varnish composed of gum dammar ten grains dissolved in one ounce of chloroform with a couple of drops of india-rubber solution to make it less brittle, any ink may be used without the addition of dextrin, should this be inadvisable.

An ordinary lantern plate when cleared with hyposulphite of sodium and washed and dried has a particularly nice surface for writing on with inks, or for coloring with a brush; in this case also dextrin is not necessary.

Ordinary photographs, and especially photographs of book illustrations, can be made much more distinct by being colored or stained. For this purpose it is necessary to have slides which are very transparent and not "fogged" in the portion through which light passes. A "thin" transparency being made apparently more dense by the staining process is often improved more than a denser one. The method consists in painting the various objects in the transparency with a brush charged with a solution of suitable aniline dye dissolved in water. If the gelatin surface of the slide be dry, the lines will be hard and spotty; it should therefore be painted while still damp. The nicest time to work is when the plate has been washed, but before it has become dry. If it has been dried it should be soaked in water for say half an hour. The surface drops of water should then be removed by a clean handkerchief or blotting-paper, and the painting proceeded with.

All aniline dyes will not answer for this purpose, as when they are mixed, in some cases, new chemical bodies are formed which have a very different color from what one would expect. Thus, while some yellows when mixed with blue produce a green color, picric acid when mixed with methylene blue gives a purplish red in the gelatin film.

The colors which I have found most satisfactory in their capabilities of being mixed, and which answer for most purposes even in painting flowers or landscapes, are eosin, chrysoidin, patent tartrazene yellow, and indigo-carmine. With these a great number of combinations of shades can be made with the same facility as an ordinary paint-box.

The gelatin takes the dye very easily,—it is only necessary to slightly tint it,—as the photograph gives the necessary light and shade. The whole operation can thus be carried out very rapidly. These dyes are very much more transparent than any of the varnish colors formerly used, infinitely more easy of application, and practically quite as permanent. To prove this latter I kept a slide colored with a number of dyes, of which one-half of each was covered with black paper, before an arc light for three hours, and while some dyes faded a good deal, the amount in those mentioned above was not noticeable, except in the case of eosin, of which the most delicate tints faded a little, but the stronger tints did not show much fading. Although these dyes undoubtedly fade by prolonged exposure to sunlight, yet the short time that a slide is allowed to remain in the lantern does not affect it at all. Many of my colored slides have been used many times during the last few years, yet appear as when first painted.

In conclusion, I hope that some of these methods will prove useful. They are all easy of application. Those mentioned first, though more or less rough, yet will be found to take the place of the black-board in most cases, which is obviously inconvenient to use when the room has been darkened, and, if there are lantern slides of other sorts to be shown, very troublesome, the lights having to be lowered and raised alternately.—*Journal of the British Dental Association.*

